# Caffeine and contraction synergistically stimulate 5′-AMP-activated protein kinase and insulin-independent glucose transport in rat skeletal muscle

**DOI:** 10.14814/phy2.12592

**Published:** 2015-10-14

**Authors:** Satoshi Tsuda, Tatsuro Egawa, Kazuto Kitani, Rieko Oshima, Xiao Ma, Tatsuya Hayashi

**Affiliations:** 1 Laboratory of Sports and Exercise Medicine, Graduate School of Human and Environmental Studies, Kyoto UniversityKyoto, 606-8501, Japan; 2 Department of Physiology, Graduate School of Health Sciences, Toyohashi SOZO UniversityToyohashi, 440-0016, Japan; 3 Key Laboratory of Puer Tea Science, Ministry of Education, Yunnan Agricultural UniversityKunming, 650201, Yunnan Province, China

**Keywords:** 5′-AMP-activated protein kinase, energy deprivation, glucose metabolism, muscle contraction, muscle fatigue

## Abstract

5′-Adenosine monophosphate-activated protein kinase (AMPK) has been identified as a key mediator of contraction-stimulated insulin-independent glucose transport in skeletal muscle. Caffeine acutely stimulates AMPK in resting skeletal muscle, but it is unknown whether caffeine affects AMPK in contracting muscle. Isolated rat epitrochlearis muscle was preincubated and then incubated in the absence or presence of 3 mmol/L caffeine for 30 or 120 min. Electrical stimulation (ES) was used to evoke tetanic contractions during the last 10 min of the incubation period. The combination of caffeine plus contraction had additive effects on AMPK*α* Thr^172^ phosphorylation, *α*-isoform-specific AMPK activity, and 3-*O*-methylglucose (3MG) transport. In contrast, caffeine inhibited basal and contraction-stimulated Akt Ser^473^ phosphorylation. Caffeine significantly delayed muscle fatigue during contraction, and the combination of caffeine and contraction additively decreased ATP and phosphocreatine contents. Caffeine did not affect resting tension. Next, rats were given an intraperitoneal injection of caffeine (60 mg/kg body weight) or saline, and the extensor digitorum longus muscle was dissected 15 min later. ES of the sciatic nerve was performed to evoke tetanic contractions for 5 min before dissection. Similar to the findings from isolated muscles incubated in vitro, the combination of caffeine plus contraction in vivo had additive effects on AMPK phosphorylation, AMPK activity, and 3MG transport. Caffeine also inhibited basal and contraction-stimulated Akt phosphorylation in vivo. These findings suggest that caffeine and contraction synergistically stimulate AMPK activity and insulin-independent glucose transport, at least in part by decreasing muscle fatigue and thereby promoting energy consumption during contraction.

## Introduction

Skeletal muscle plays a major role in whole-body glucose metabolism in rodents and humans. Insulin and exercise (muscle contraction) are the physiologically important stimuli of glucose transport across the cell membrane, which is the rate-limiting step in glucose utilization in skeletal muscle. Although both insulin and exercise elicit the translocation of glucose transporter 4 (GLUT4) from intracellular vesicle compartments to the sarcolemma and T-tubules, these stimuli activate specific signaling mechanisms. 5′-Adenosine monophosphate-activated protein kinase (AMPK) has been identified as a signaling molecule involved in contraction-stimulated and insulin-independent glucose transport (reviewed in Fujii et al. 2006; Friedrichsen et al. 2013). AMPK in skeletal muscle has also been implicated in a number of the metabolic effects of exercise such as increased insulin sensitivity (Fisher et al. 2002; Iglesias et al. 2002) and GLUT4 expression (Zheng et al. 2001; Nakano et al. 2006), inhibition of acetyl-CoA carboxylase and fatty acid oxidation (Winder and Hardie 1996; Vavvas et al. 1997), modulation of glycogen synthesis (Miyamoto et al. 2007; Hunter et al. 2011), mitochondrial biogenesis via peroxisome proliferator-activated receptor-*γ* coactivator 1*α* (PGC1*α*) (Jager et al. 2007), activation of sirtuin (Canto et al. 2009), and the shift in the metabolic properties toward those of slow oxidative muscle fibers (Rockl et al. 2007). These acute and chronic alterations in skeletal muscle suggest that AMPK is a metabolic enhancer that may prevent or delay the development of type 2 diabetes mellitus.

AMPK is a heterotrimer comprising a catalytic *α* subunit, and regulatory *β* and *γ* subunits. There are two different *α* isoforms (*α*1 and *α*2) (Stapleton et al. 1996); *α*1 is expressed ubiquitously and *α*2 is expressed in skeletal muscle, heart, and liver. AMP binding results in the phosphorylation of *α* Thr^172^, which is essential for kinase activation (Stein et al. 2000). Classically, AMPK acts as a signaling intermediary by monitoring cellular energy status (Hardie 2011). In isolated rat skeletal muscle incubated in vitro, both *α*1-containing AMPK (AMPK*α*1) and *α*2-containing (AMPK*α*2) are stimulated by energy-decreasing (AMP-increasing) stressors including contraction, hypoxia, chemical inhibition of oxidative phosphorylation, and hyperosmolarity, all of which are potent stimulators of insulin-independent glucose transport (Hayashi et al. 2000).

Caffeine (1,3,7-trimethylxanthine) has been implicated in the activation of insulin-independent glucose transport in rodent skeletal muscles (Wright et al. 2004; Jensen et al. 2007; Abbott et al. 2009; Egawa et al. 2009, 2011a), and AMPK has been considered as a signaling intermediary involved in the metabolic activation by caffeine (Wright et al. 2004; Jensen et al. 2007; Raney and Turcotte 2008; Abbott et al. 2009; Egawa et al. 2009, 2011a). We reported previously that incubation with caffeine (≥3 mmol/L for ≥15 min) increased AMPK*α* Thr^172^ phosphorylation and AMPK*α*1 and *α*2 activities in isolated rat skeletal muscles and that these effects were accompanied by increased insulin-independent glucose transport (Egawa et al. 2009). Caffeine-induced AMPK activation was also accompanied by decreased fuel status; for example, the phosphocreatine (PCr) content was 23% lower in muscle stimulated with caffeine compared with the control (Egawa et al. 2009). These results indicated that caffeine acts directly in skeletal muscle and has similar actions to those of contraction by acutely promoting AMPK activity with energy deprivation in skeletal muscle. It is notable that epidemiological studies have demonstrated that the intake of caffeinated beverages, including coffee and tea, is linked to a reduced risk of type 2 diabetes mellitus (Huxley et al. 2009; Ding et al. 2014).

Caffeine increases force production during contraction by multiple mechanisms such as increased Ca^2+^ release and Ca^2+^ permeability in the sarcoplasmic reticulum, increased Ca^2+^ sensitivity, and slowing of the sarcoplasmic reticulum Ca^2+^ pump (reviewed in Allen and Westerblad 1995; Magkos and Kavouras 2005). Many researchers have reported that caffeine increases exercise performance and delays fatigue in rodents (Ryu et al. 2001; Zheng et al. 2014) and humans (Costill et al. 1978; Graham and Spriet 1995; Tarnopolsky and Cupido 2000; Ryu et al. 2001; Simmonds et al. 2010). These ergogenic actions of caffeine led us to hypothesize that caffeine stimulates AMPK and glucose transport in contracting states by causing profound changes in the cellular energy status in skeletal muscle. To test our hypothesis, we examined the effect of caffeine stimulation on isolated rat skeletal muscle electrically stimulated in vitro. We also explored the effect of systemic caffeine administration on contracting skeletal muscle in living rats.

## Materials and Methods

### Animals

Male Sprague Dawley rats (150–160 g) were purchased from Shimizu Breeding Laboratories (Kyoto, Japan). Rats were fed a standard diet (Certified Diet MF; Oriental Koubo, Tokyo, Japan) and water ad libitum, and fasted overnight before each experiment. Protocols for animal use and euthanasia were approved by the Kyoto University Graduate School of Human and Environmental Studies and Kyoto University Radioisotope Research Center.

### Muscle treatment in vitro

Muscles were treated as we described previously (Toyoda et al. 2004) with some modifications. Rats were killed by cervical dislocation, and the epitrochlearis muscle (Nesher et al. 1980) was removed and mounted on an incubation apparatus with a tension set to 0.5 g. The muscle was preincubated in 7 mL of alpha minimum essential medium (*α*MEM) containing 1% penicillin/streptomycin for 40 min. The muscle was then incubated in 7 mL of fresh medium in the absence or presence of 3 mmol/L caffeine for 30 or 120 min, 1 mmol/L caffeic acid for 30 min, or 1 mmol/L chlorogenic acid for 30 min. These reagents did not affect resting tension during the incubation period. For tetanic contraction, the muscle was stimulated with an electric stimulator (SEN-3401; Nihon Kohden, Tokyo, Japan) during the last 10 min of the incubation period (train rate: 1/min, train duration: 10 sec, pulse rate: 100 Hz, pulse duration: 0.1 msec, voltage: 10 V). Force was recorded with a force transducer (TRN001; Kent Scientific, Torrington, CT) and a recorder (U-228-2P-500; Pantos, Kyoto, Japan). Control muscles were preincubated and incubated without contraction. Other muscle samples were used fresh in the 3-*O*-methyl-d-glucose (3MG) transport or caffeine transport assay, or were immediately frozen in liquid nitrogen and subjected to western blot analysis and assays to measure isoform-specific AMPK activity, and ATP and PCr contents. All media were gassed with 95% O_2_/5% CO_2_ and maintained at 37°C.

### Muscle treatment in vivo

Caffeine dissolved in saline was injected intraperitoneally without anesthesia at 60 mg/kg body weight. The injection volume was 2 mL/kg body weight. Five minutes after caffeine or saline injection, rats were anesthetized with intraperitoneal administration of pentobarbital sodium (75 mg/kg body weight), and electrodes (OM209-041; Unique Medical, Tokyo, Japan) were attached to the sciatic nerve on both sides. Fifteen minutes after caffeine or saline injection, the extensor digitorum longus (EDL) muscle was rapidly dissected. The muscle was used fresh to measure 3MG transport activity or other samples were immediately frozen in liquid nitrogen and subjected to western blot analysis or the assay to measure isoform-specific AMPK activity. For tetanic contraction, the sciatic nerves were stimulated during the last 5 min before dissection (train rate: 1/min, train duration: 10 sec, pulse rate: 100 Hz, pulse duration: 0.1 msec, voltage: 2 V) using the SEN-3401 stimulator.

### Western blot analysis

Western blot analysis was performed as we described previously (Toyoda et al. 2004) with some modifications. The muscle was homogenized in ice-cold lysis buffer (1:40 wt/vol) containing 20 mmol/L Tris HCl (pH 7.4), 1% Triton X-100, 50 mmol/L NaCl, 250 mmol/L sucrose, 50 mmol/L NaF, 5 mmol/L sodium pyrophosphate, 2 mmol/L dithiothreitol, 4 mg/L leupeptin, 50 mg/L trypsin inhibitor, 0.1 mmol/L benzamidine, and 0.5 mmol/L phenylmethylsulfonyl fluoride (buffer A) and centrifuged at 16,000*g* for 40 min at 4°C. Denatured proteins were separated on a polyacrylamide gel and transferred to a polyvinylidene difluoride membrane. The membrane was blocked with TBS-T (TBS with 0.1% Tween 20) containing 5% nonfat dry milk and was then incubated with commercially available antibodies (AMPK*α* Thr^172^ [#2531], AMPK*α* [#2532], Akt Ser^473^ [#9271], Akt [#9272]; all from Cell Signaling Technology, Beverly, MA). Some membranes were incubated with a signal enhancer (Can Get Signal® Immunoreaction Enhancer Solution; Toyobo Co., Ltd., Tokyo, Japan). The membrane was then washed and incubated with anti-rabbit IgG coupled to peroxidase. Protein signals were developed with enhanced chemiluminescence reagents (GE Healthcare, Buckinghamshire, U.K.), detected with ImageCapture G3 (Liponics, Tokyo, Japan), and quantified with ImageJ software (Abramoff et al. 2004). The mean signal intensity of the control samples for each membrane was used as a reference for correcting gel-to-gel variation.

### Isoform-specific AMPK activity assay

AMPK activity was measured as we described previously (Toyoda et al. 2004). The muscle was homogenized in buffer A, and the supernatant (100 *μ*g of protein) was immunoprecipitated with the *α*1 or *α*2 antibody and protein A-Sepharose (GE Healthcare). The kinase reaction was performed with the synthetic SAMS peptide as the substrate. The kinase activity is expressed as incorporated ATP per minute per milligram of immunoprecipitated protein. AMPK*α*1 and AMPK*α*2 activities account for 20–30% and 70–80% of basal AMPK activity, respectively, in rat skeletal muscle (Cheung et al. 2000). The *α*2-isoform is also the major *α*-isoform in human vastus lateralis muscle (Wojtaszewski et al. 2005).

### Caffeine transport

After preincubation, the epitrochlearis muscle was incubated in 7 mL of *α*MEM containing 3 mmol/L [^14^C] caffeine (0.3 *μ*Ci/mL) and 1 mmol/L d-[1-^3^H] mannitol (1.5 *μ*Ci/mL) (both from American Radiolabeled Chemicals, St. Louis, MO) at 37°C for up to 120 min with or without contraction during the last 10 min. Control muscle was preincubated and incubated in 7 mL of *α*MEM containing 3 mmol/L [^14^C] caffeine (0.3 *μ*Ci/mL) and 1 mmol/L d-[1-^3^H] mannitol (1.5 *μ*Ci/mL) at 37°C for 10 sec. The muscle was blotted onto filter paper, trimmed, and frozen in liquid nitrogen. The muscle was then digested in 1 mol/L NaOH at 80°C for 10 min and the reaction neutralized with 1 mol/L HCl. The particulates were precipitated by centrifugation at 20,000*g* for 3 min, and the radioactivity in the supernatant was measured in a liquid scintillation counter. The volume of intracellular space was calculated as described previously (Young et al. 1986), and the intracellular caffeine concentration was calculated.

### 3MG transport

The 3MG transport assay was performed as we described previously (Toyoda et al. 2004). For muscle treatment in vitro, after the incubation period, the epitrochlearis muscle was incubated in 2 mL of transport buffer comprising Krebs–Ringer buffer (KRB) containing 1 mmol/L [^3^H]3-MG (1.5 *μ*Ci/mL) and 7 mmol/L d-[1-^14^C] mannitol (0.3 *μ*Ci/mL) (both from American Radiolabeled Chemicals) at 30°C for 10 min. For muscle treatment in vivo, the dissected EDL muscle was preincubated for 10 min in 7 mL of KRB containing 2 mmol/L sodium pyruvate and then incubated in 2 mL of transport buffer for 10 min. The muscle sample was then treated as described in Caffeine transport section earlier. The transport activity is expressed as the amount of 3MG taken up per volume of intracellular space per hour.

### ATP, PCr, and glycogen assay

ATP, PCr, and glycogen contents were measured enzymatically as we described previously (Nakano et al. 2006; Egawa et al. 2009). The values are expressed as nanomoles of ATP, PCr, and glucosyl unit per milligram wet weight of muscle, respectively.

### Statistical analysis

The results are presented as mean ± SE. Multiple means were compared using analysis of variance (ANOVA) followed by post hoc comparisons with Tukey’s test. Two means were compared using unpaired Student’s *t* test. Differences between groups were considered significant at *P *<* *0.05.

## Results

### Caffeine and contraction additively stimulate AMPK in isolated skeletal muscle

**Figure 1:**
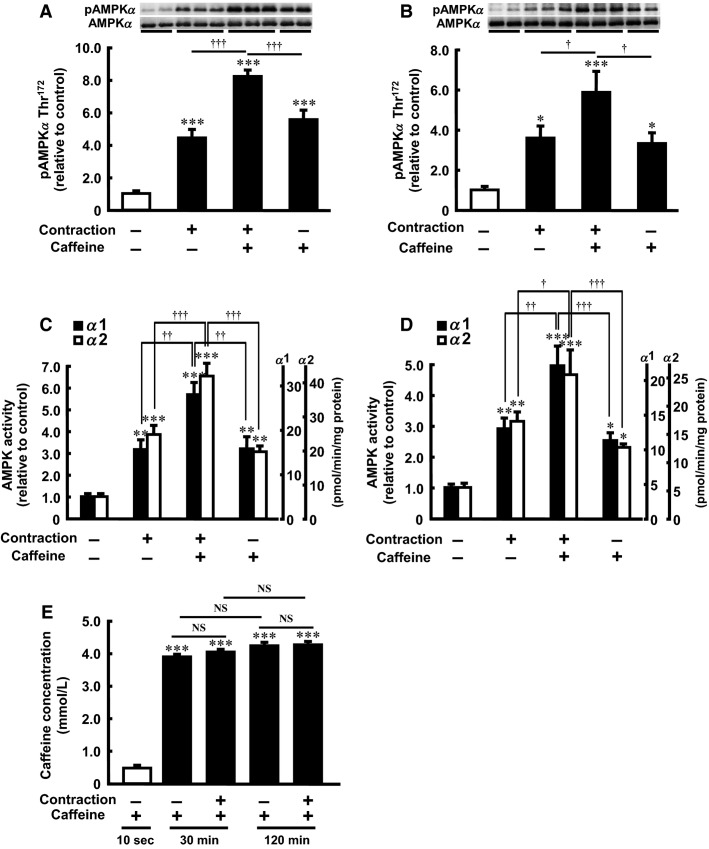
Effect of caffeine on contraction-stimulated AMPK*α* Thr^172^ phosphorylation and AMPK activity in incubated rat skeletal muscle. Isolated epitrochlearis muscle was preincubated and incubated for 30 min (A and C) or 120 min (B and D) in the absence (−) or presence (+) of 3 mmol/L caffeine. The muscle was tetanically contracted during the last 10 min of the incubation period and then subjected to western blot analysis (A and B) or an isoform-specific AMPK activity assay (C and D). Intracellular caffeine concentration was measured after incubation in the presence of 3 mmol/L caffeine for up to 120 min with or without electrical stimulation (E). Values are mean ± SE; *n* = 5–10 per group. *P <* *0.05, **P <* *0.01, ***P <* *0.001 versus control; ^†^*P *<* *0.05, ^††^*P *<* *0.01, ^†††^*P *<* *0.001 versus contraction plus caffeine. AMPK*α*, 5′-Adenosine monophosphate-activated protein kinase *α*; NS, not significant.

Our previous study demonstrated that maximal activation of AMPK by caffeine is observed with a 30-min incubation at a concentration of 3 mmol/L (Egawa et al. 2009) and that maximal activation of AMPK by contraction can be induced by 10 repeated 10 sec tetanic contractions during 10 min (Musi et al. 2001). In the present study, the stimulatory effects of maximally effective caffeine and maximally effective contraction on AMPK*α* Thr^172^ phosphorylation were partly but significantly additive (Fig.1A). A combined effect on AMPK*α* Thr^172^ phosphorylation was also found after incubation with caffeine for 120 min (Fig.1B). The total AMPK content did not differ between the groups. The caffeine- and contraction-stimulated AMPK*α*1 and AMPK*α*2 activities were also significantly additive (Fig.1C and D). In the caffeine transport assay, the intracellular concentration of caffeine reached a maximum by 30 min and was maintained at this level at 120 min after the start of the exposure to caffeine. Contraction did not affect the intracellular caffeine concentration (Fig.1E).

### Caffeine and contraction additively stimulate glucose transport in isolated skeletal muscle

**Figure 2:**
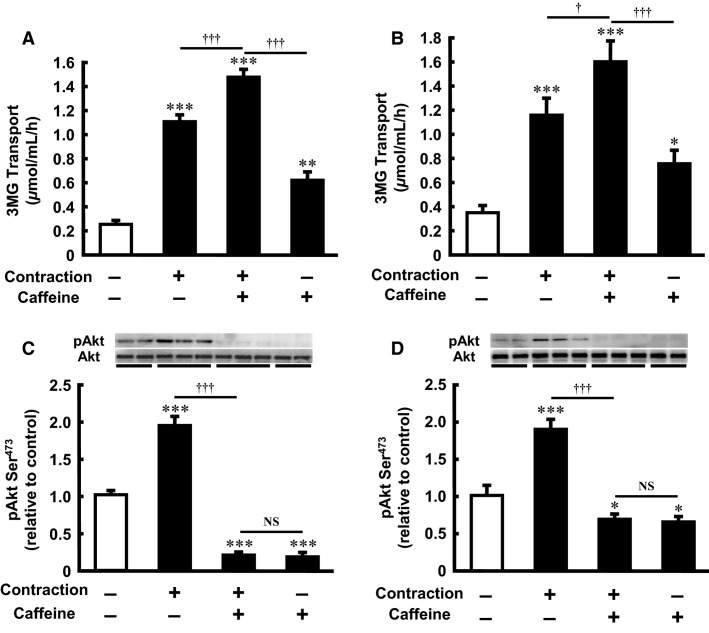
Effect of caffeine on contraction-stimulated 3-*O*-methyl-d-glucose (3MG) transport activity and Akt Ser^473^ phosphorylation in incubated rat skeletal muscle. Isolated epitrochlearis muscle was preincubated and incubated for 30 (A and C) or 120 (B and D) min in the absence (−) or presence (+) of 3 mmol/L caffeine. The muscle was tetanically contracted during the last 10 min of the incubation period and then subjected to the 3MG transport assay (A and B) or western blot analysis (C and D). Values are mean ± SE; *n* = 5–13 per group. *P <* *0.05, **P <* *0.01, ***P <* *0.001 versus control; ^†^*P *<* *0.05, ^†††^*P *<* *0.001 versus contraction plus caffeine; NS, not significant.

Our previous study demonstrated that maximal activation of glucose transport by contraction can be induced by 10 repeated 10 sec tetanic contractions (Musi et al. 2001). Treatment with 3 mmol/L caffeine for 30 min and contraction increased the rate of 3MG transport by 2.4- and 4.4-fold compared with the basal level, respectively. The caffeine- and contraction-stimulated activity of 3MG transport was significantly additive (5.8-fold compared with the basal level) (Fig.2A). Similarly, treatment with 3 mmol/L caffeine for 120 min and contraction increased the rate of 3MG transport by 2.2- and 3.3-fold compared with the basal level, respectively. The caffeine- and contraction-stimulated activity of 3MG transport was significantly additive (4.6-fold compared with the basal level) (Fig.2B).

### Caffeine inhibits basal and contraction-stimulated Akt Ser^473^ phosphorylation in isolated skeletal muscle

Contraction increases the phosphorylation of Akt Ser^473^ in rat skeletal muscle (Sakamoto et al. 2002). In our previous study (Iwanaka et al. 2010), tetanic contraction significantly increased Ser^473^ phosphorylation of Akt in isolated rat epitrochlearis muscle. On the other hand, we have also shown that caffeine (3 mmol/L, 15 min) decreases the basal and insulin-stimulated phosphorylation of Akt in isolated rat epitrochlearis muscle (Egawa et al. 2011b). In the present study, contraction significantly increased Akt phosphorylation, but caffeine (3 mmol/L, 30 min) markedly inhibited basal and contraction-stimulated Akt Ser^473^ phosphorylation (Fig.2C). This effect of caffeine on Akt Ser^473^ phosphorylation in skeletal muscle persisted after incubation with caffeine for 120 min (Fig.2D). The total Akt content did not differ between the groups.

### Caffeine and contraction additively decrease ATP and PCr contents in isolated skeletal muscle

**Figure 3:**
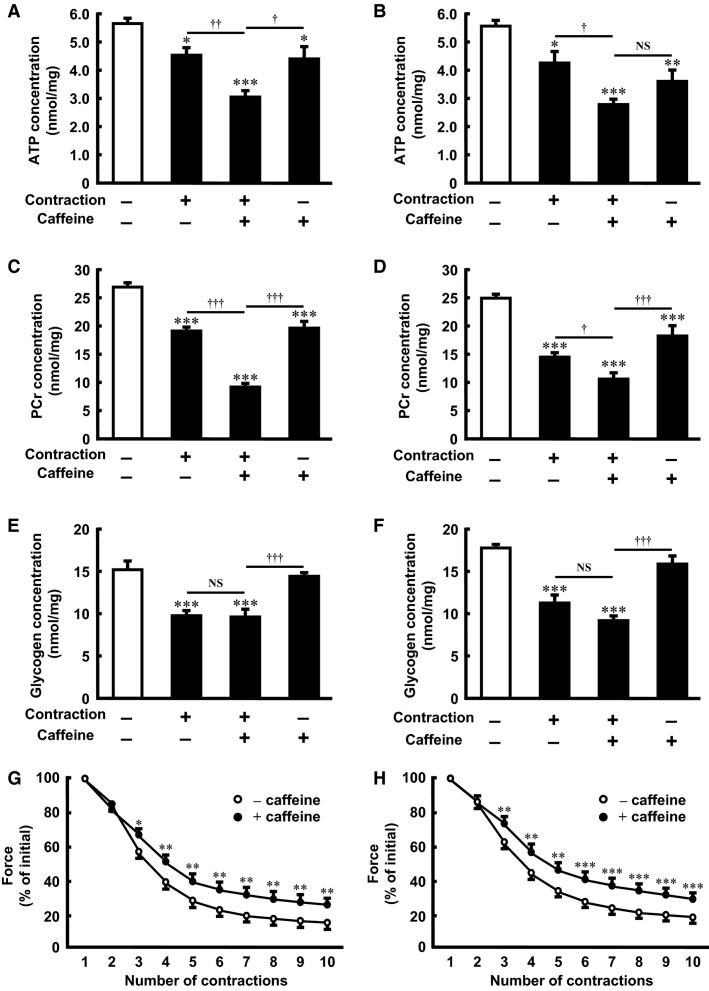
Effect of caffeine on ATP, PCr, and glycogen contents, and force production during contraction in incubated rat skeletal muscle. Isolated epitrochlearis muscle was preincubated and incubated for 30 min (A, C, E, and G) or 120 min (B, D, F, and H) in the absence (−) or presence (+) of 3 mmol/L caffeine. The muscle was tetanically contracted during the last 10 min of the incubation period, after which the concentrations of ATP (A and B), PCr (C and D), and glycogen (E and F) were measured. Peak tension of each contraction was evaluated (G and H). Values are mean ± SE; *n* = 4–12 per group. *P <* *0.05, **P <* *0.01, ***P <* *0.001 versus control; ^†^*P *<* *0.05, ^††^*P *<* *0.01, ^†††^*P *<* *0.001 versus contraction plus caffeine. PCr, phosphocreatine; NS, not significant.

To clarify whether the combined effect of caffeine and contraction on AMPK activity is associated with a change in energy status, we measured the muscle contents of ATP, PCr, and glycogen. Treatment with 3 mmol/L caffeine for 30 or 120 min and contraction decreased the contents of ATP (Fig.3A and B) and PCr (Fig.3C and D). The effects of caffeine and contraction on ATP and PCr were partially additive (Fig.3A–D). Consistent with these findings, caffeine significantly mitigated muscle fatigue during contraction (Fig.3G and H), in association with an increase in the initial peak force (30-min caffeine treatment [239 ± 11 mN, *n* = 8] vs. control [188 ± 21 mN, *n* = 7]; *P* < 0.05). Treatment with caffeine did not affect the content of glycogen (Fig.3E and F).

### Neither caffeic acid nor chlorogenic acid affects contraction-stimulated AMPKα Thr^172^ phosphorylation in isolated skeletal muscle

**Figure 4:**
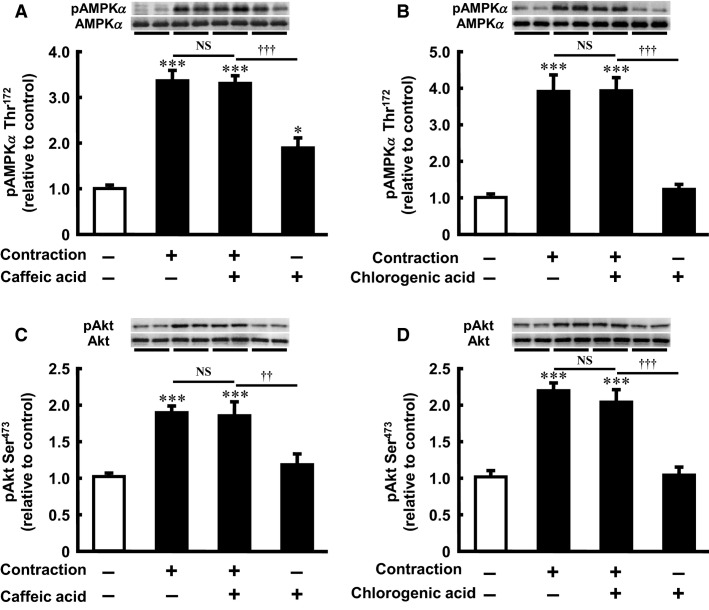
Effect of caffeic acid and chlorogenic acid on AMPK*α* Thr^172^ phosphorylation and Akt Ser^473^ phosphorylation in incubated rat skeletal muscle. Isolated epitrochlearis muscle was preincubated and incubated for 30 min in the absence (−) or presence (+) of 1 mmol/L caffeic acid (A and C) or 1 mmol/L chlorogenic acid (B and D). The muscle was tetanically contracted during the last 10 min of the incubation period and then subjected to western blot analysis. Values are mean ± SE; *n* = 5–12 per group. *P <* *0.05, ***P <* *0.001 versus control; ^††^*P *<* *0.01, ^†††^*P *<* *0.001 versus contraction plus caffeic acid or chlorogenic acid. AMPK*α*, 5′-Adenosine monophosphate-activated protein kinase *α*; NS, not significant.

In addition to caffeine, caffeic acid and chlorogenic acid, which are the major constituents of coffee, also have antihyperglycemic properties (Hsu et al. 2000; Cheng et al. 2003; Jung et al. 2006; Bassoli et al. 2008). We previously reported that caffeic acid, but not chlorogenic acid, acutely promoted AMPK phosphorylation. In that study, the maximal activation of AMPK by caffeic acid was observed at 1 mmol/L after a 30-min incubation in isolated rat epitrochlearis muscle (Tsuda et al. 2012). In the current study, we examined the effects of caffeic acid and chlorogenic acid on contraction-stimulated AMPK activity in skeletal muscle. AMPK*α* Thr^172^ phosphorylation was increased by caffeic acid (1 mmol/L, 30 min) (Fig.4A), but not by chlorogenic acid (1 mmol/L, 30 min) (Fig.4B). However, unlike caffeine (Fig.2A and B), incubation with caffeic acid or chlorogenic acid did not affect the contraction-stimulated AMPK*α* Thr^172^ phosphorylation (Fig.4A and B). The total AMPK content did not differ between the groups.

### Neither caffeic acid nor chlorogenic acid inhibits basal and contraction-stimulated Akt Ser^473^ phosphorylation in isolated skeletal muscle

We previously reported that caffeic acid and chlorogenic acid had no effect on Akt Ser^473^ phosphorylation in isolated rat epitrochlearis muscle (Tsuda et al. 2012). In the present study, unlike caffeine (Fig.2C and D), contraction-stimulated Akt Ser^473^ phosphorylation was not affected by incubation with caffeic acid or with chlorogenic acid (Fig.4C and D). The total Akt content did not differ between the groups.

### Intraperitoneal caffeine injection and contraction in situ additively activate AMPK and glucose transport in skeletal muscle

**Figure 5:**
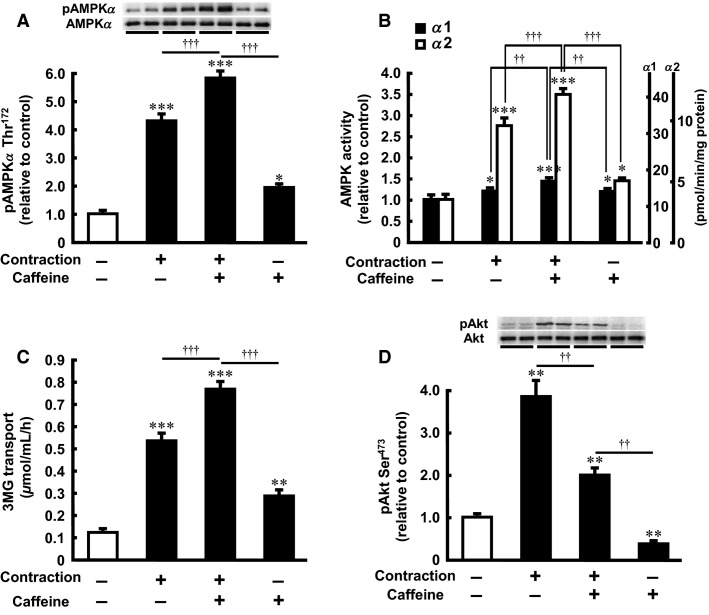
Effect of intraperitoneal caffeine injection on contraction-stimulated AMPK*α* Thr^172^ phosphorylation, AMPK activity, 3MG transport activity, and Akt Ser^473^ phosphorylation in rat skeletal muscle. Caffeine (60 mg/kg) or saline was injected intraperitoneally. Fifteen minutes after injection of caffeine or saline, the EDL was dissected and subjected to western blot analysis (A and D), isoform-specific AMPK activity assay (B), or 3MG transport assay (C). Tetanic contraction was elicited by electrical stimulation of the sciatic nerve during the last 5 min before dissection. Representative immunoblots are shown. Values are mean ± SE; *n* = 4–11 per group. *P <* *0.05, **P <* *0.01, ***P <* *0.001 versus control; ^††^*P *<* *0.01, ^†††^*P *<* *0.001 versus contraction plus caffeine. AMPK*α*, 5′-Adenosine monophosphate-activated protein kinase *α*; 3MG, 3-*O*-methyl-d-glucose; EDL, extensor digitorum longus.

To determine whether caffeine affects contraction-stimulated AMPK activity in vivo, we measured the degree of phosphorylation of AMPK*α* Thr^172^ in EDL muscle dissected after intraperitoneal injection of caffeine or saline with or without contraction. AMPK phosphorylation was increased by caffeine and contraction, and the effects of caffeine and contraction were partially additive (Fig.5A). The total AMPK content did not differ between the groups. In the isoform-specific AMPK activity assay, caffeine and contraction increased both AMPK*α*1 and AMPK*α*2 activities, and the effects of caffeine and contraction were partially additive (Fig.5B). Caffeine injection and contraction also increased 3MG transport by 2.3- and 4.3-fold compared with the saline injection, respectively (Fig.5C). The effects of caffeine and contraction were partially additive (6.2-fold compared with the basal level) (Fig.5C). Similarly, Akt Ser^473^ phosphorylation was significantly increased by contraction, and caffeine significantly suppressed contraction-stimulated Akt Ser^473^ phosphorylation (Fig.5D). The total Akt content did not differ between the groups.

## Discussion

One important finding of the present study is that the combination of the maximally effective caffeine concentration and maximally effective contraction increased AMPK phosphorylation and both *α*1 and *α*2 activities more than either of the stimuli alone. These results suggest that caffeine increases the maximal capacity of contraction-stimulated AMPK activation in skeletal muscle. We have demonstrated previously that 10 repeated 10 sec tetanic contractions can induce maximal AMPK activity in incubated rat epitrochlearis muscle and that there is no further increase in AMPK activity with 15 repeated 10 sec tetanic contractions (Musi et al. 2001). However, tetanic contraction is not the strongest stimulus of AMPK activity in skeletal muscle. For instance, in incubated rat epitrochlearis muscle, dinitrophenol (0.5 mmol/L for 20 min) increased AMPK activity by sixfold compared with basal AMPK activity, and 10 sec tetanic contractions increased AMPK*α*2 activity only fourfold (Hayashi et al. 2000). Therefore, even when AMPK activity is increased maximally by contraction in skeletal muscle, it may still be activated further by other stimuli.

Another important finding is that incubation with caffeine ameliorated muscle fatigue during contraction. Caffeine can easily pass through the surface membrane of the muscle cell because of its hydrophobic property (Bianchi 1962). In this study, the intracellular concentration of caffeine increased rapidly after exposure to caffeine and was not affected by contraction (Fig.1E). The ergogenic actions of caffeine may contribute to the decreased muscle fatigue and profound decrease in energy status in contracting skeletal muscle. However, energy deprivation may not be essential for increasing contraction-stimulated AMPK activity and glucose transport. We showed previously that contraction-stimulated 3MG transport increased significantly in the presence of leucine in isolated rat epitrochlearis muscle (Iwanaka et al. 2010). In that study, the basal 3MG transport was unaffected by leucine (2 mmol/L, 30 min), but contraction-stimulated 3MG transport was increased by 24% by leucine. The basal phosphorylation of AMPK was not altered by leucine, but leucine significantly promoted contraction-stimulated phosphorylation. We also measured the basal and contraction-stimulated levels of ATP and PCr, but leucine had no effect on these parameters. Correspondingly, leucine had no effect on muscle fatigue during contraction. Thus, caffeine may stimulate AMPK via both energy-dependent and -independent mechanisms in contracting skeletal muscle.

In the mechanism of energy reduction by caffeine, Miyazaki et al. (1962) demonstrated that 1–5 mmol/L of caffeine increased oxygen consumption acutely in frog skeletal muscles that were isolated and incubated in vitro. Those authors also found that the metabolic enhancement afforded by caffeine was associated with an increase in lactic acid content and decreases in ATP, ADP, and PCr contents in the muscle, without mechanical changes such as contracture formation. Thus, caffeine may act on the muscle energy status via acceleration of the energy supply, rather than via inhibition of mitochondrial function and suppression of ATP production.

We previously showed that caffeic acid (≥0.1 mmol/L, ≥30 min) promoted AMPK phosphorylation in resting isolated rat epitrochlearis muscle (Tsuda et al. 2012). This effect was associated with increased 3MG transport and decreased PCr level. In the present study, unlike caffeine, the combination of the maximally effective caffeic acid and tetanic contraction was not additive in AMPK phosphorylation. These data show clearly that AMPK-activating agents do not necessarily have an additive effect on contraction-stimulated AMPK activity in skeletal muscle.

We found that the stimulatory effect of contraction on Akt was abolished by caffeine (Fig.2C and D). By contrast, the stimulatory effect of contraction on Akt was not affected by caffeic acid or chlorogenic acid (Fig.4C and D). These data raise the possibility that inhibition of Akt activity may play a stimulatory or permissive role in contraction-stimulated AMPK activation in skeletal muscle. However, Akt2 knockout does not affect treadmill exercise-stimulated 2-deoxuglucose transport in mouse soleus muscle or contraction-stimulated AMPK phosphorylation in mouse tibialis anterior muscle (Sakamoto et al. 2006). Furthermore, pharmacological inhibition of contraction-stimulated Akt phosphorylation by wortmannin does not affect contraction-stimulated AMPK phosphorylation or 3MG transport in isolated rat epitrochlearis muscle (Funai and Cartee 2009). Thus, it is unlikely that decreased Akt activity per se leads to AMPK activation in contacting skeletal muscle.

Contraction and insulin use distinct signaling molecules, and insulin-stimulated Akt is an important mediator of glucose transport and glycogen synthesis in skeletal muscle (reviewed in Fujii et al. 2006; Wu et al. 2011). On the other hand, contraction-stimulated Akt has been implicated in the activation of protein synthesis and increase in muscle mass (Wu et al. 2011). Further studies are needed to investigate the effects of caffeine on glucose phosphorylation or on pathways that act downstream of glucose phosphorylation, such as glycolysis/oxidative phosphorylation and glycogen synthesis. However, the glucose molecules transported into caffeine-stimulated muscle cells might be used as an energy source for continuing contraction, rather than as an energy source for facilitating protein synthesis or as substrate for glycogen synthesis.

Caffeine has been shown to inhibit insulin-stimulated signaling, including Akt in skeletal muscles (Foukas et al. 2002; Kolnes et al. 2010; Egawa et al. 2011b). We have reported that caffeine (3 mmol/L) decreases both the basal and insulin-stimulated phosphorylation of the insulin receptor substrate 1 (IRS-1) Tyr^612^, Akt Ser^473^, and glycogen synthase kinase (GSK) *β* Ser^9^, as well as insulin-stimulated 3MG transport, without affecting insulin receptor tyrosine phosphorylation in isolated rat epitrochlearis muscle. Correspondingly, caffeine promotes the inhibitory phosphorylation of IRS-1 (Ser^307^), and enhances the stimulatory phosphorylation of an IRS-1 Ser^307^ kinase, the inhibitor-*κ*B kinase (IKK) *α*/*β* (Ser^176/180^) (Egawa et al. 2011b). These effects of caffeine on IRS-1 and Akt have been also observed after intravenous administration of caffeine to living rats at a physiological dose (5 mg/kg) (Egawa et al. 2011b). On the other hand, contraction-induced Akt activation is not associated with the activation of insulin receptor or IRS-1, and is inhibited by pharmacological blockade of phosphatidylinositol 3-kinase (PI3K) using wortmannin and LY294002 (Sakamoto et al. 2002). However, the precise mechanism of contraction-induced Akt activation remains to be elucidated. It is notable that caffeine at a high concentration (10 mmol/L), which is sufficient to induce muscle contracture, inhibits not only insulin-stimulated, but also contraction-stimulated glucose transport in isolated rat soleus muscle (Kolnes et al. 2010). Although further studies are required to elucidate this issue, caffeine at a high concentration may play a direct and/or indirect antagonistic role regarding the molecule(s) that lead to GLUT4 translocation in skeletal muscle.

In conclusion, we provide for the first time evidence suggesting that caffeine and contraction synergistically increase AMPK activity and insulin-independent glucose transport and concomitantly decrease energy status and muscle fatigue in skeletal muscle. The ergogenicity of caffeine may contribute to the increase in AMPK activity in contracting skeletal muscle, even though contraction and caffeine are each strong activators of AMPK.
